# Supplementary material for: A Likelihood Approach for Real-Time Calibration of Stochastic Compartmental Epidemic Models
Source: PLoS Comput Biol. 2017 Jan 17;13(1):e1005257. doi: 10.1371/journal.pcbi.1005257 (PMC5240920; doi:10.1371/journal.pcbi.1005257)
Supplement: S1 File — (TAR.GZ) [file pcbi.1005257.s014.tar.gz › HSPH_Online-SI-Revision/PF08/n1-extreme/n1-extreme_PF_table-4.pdf]

1	R0	{2.95019, 14.2854}	{1.79151, 13.0348}	{{1.07874, 6.77341}, {2.7811, 18.834}}	{39.3259, 24.1795}	inf	56.	77.79	{5.87089, 186.869}	98.1286	0.455169	newly	31.	44.88	{4., 101.}	99.2903	0.433727	Reff	2.70237	1.34314	{0.894697, 2.0885}	50.2979	ar	924.	610.73	{175., 914.}	34.1786
2	R0	{2.67565, 16.946}	{1.77921, 13.4164}	{{1.11927, 6.62607}, {2.67299, 18.9003}}	{34.3417, 25.5238}	inf	194.	80.8152	{6.0459, 161.186}	61.2473	0.622371	newly	79.	38.56	{6., 74.}	53.8987	0.529483	Reff	1.69101	1.06829	{0.656701, 1.61873}	41.1375	ar	926.	632.63	{260., 902.}	31.8132
3	R0	{2.40066, 16.6543}	{1.79052, 13.0902}	{{1.11461, 6.31117}, {2.60082, 18.8445}}	{29.7834, 26.6631}	inf	86.	82.4073	{6.60572, 204.627}	69.8554	0.483155	newly	43.	41.05	{6., 96.}	59.6512	0.404805	Reff	2.02856	1.20467	{0.77803, 1.77856}	43.7333	ar	916.	620.32	{226., 908.}	32.5633
4	R0	{1.9294, 15.4361}	{1.69607, 13.6044}	{{1.11228, 5.72868}, {2.24606, 19.0443}}	{23.5349, 26.1428}	inf	96.	77.0495	{2.48733, 170.612}	61.4635	0.541362	newly	51.	38.95	{1., 83.}	53.3529	0.480028	Reff	1.6207	1.15378	{0.783675, 1.59714}	32.6546	ar	796.	606.23	{245., 866.}	27.9761
5	R0	{2.92859, 19.5393}	{1.55874, 13.3725}	{{1.1993, 4.60339}, {2.09202, 20.}}	{46.8488, 32.2916}	inf	172.	64.7226	{8.29412, 128.972}	65.1874	0.619383	newly	68.	29.37	{5., 55.}	58.7206	0.498118	Reff	1.94751	0.893988	{0.649667, 1.19193}	54.0959	ar	927.	692.12	{473., 936.}	26.302
6	R0	{1.79622, 12.3148}	{1.71972, 13.4094}	{{1.14096, 5.72868}, {2.24606, 18.9248}}	{22.9053, 34.8577}	inf	52.	78.1316	{2.57648, 164.906}	110.249	0.516465	newly	27.	36.07	{3., 77.}	84.7778	0.442848	Reff	1.38489	0.981093	{0.640215, 1.51022}	34.3108	ar	734.	605.26	{238., 869.}	28.2153
7	R0	{2.45855, 17.1776}	{1.83181, 13.4103}	{{1.11228, 6.31117}, {2.73737, 18.9813}}	{29.4927, 25.788}	inf	118.	91.2379	{8.98592, 227.207}	64.4317	0.511824	newly	59.	47.39	{5., 97.}	57.0678	0.448474	Reff	1.87833	1.23918	{0.814482, 1.91401}	37.5647	ar	871.	643.98	{276., 905.}	27.8599
8	R0	{2.20009, 15.519}	{1.75456, 14.259}	{{1.22062, 8.26756}, {2.40871, 20.}}	{26.1038, 23.8734}	inf	146.	88.7725	{26.9013, 180.377}	54.2176	0.398121	newly	70.	40.84	{14., 80.}	50.7429	0.351898	Reff	1.44546	1.00665	{0.633401, 1.42161}	32.2255	ar	839.	782.	{507., 1019.}	18.1478
9	R0	{2.06493, 9.6414}	{1.83118, 13.1276}	{{1.11228, 6.74711}, {2.79269, 18.8445}}	{27.8933, 48.4857}	inf	38.	83.0598	{11.0203, 188.089}	156.432	0.459338	newly	41.	47.34	{5., 98.}	74.0488	0.405678	Reff	1.89148	1.34711	{0.872175, 2.0885}	33.2654	ar	777.	634.59	{234., 914.}	28.583
10	R0	{2.60775, 19.6936}	{1.67705, 15.3051}	{{1.19222, 7.91725}, {2.3882, 20.}}	{37.2294, 22.9261}	inf	154.	95.9539	{32.6247, 211.004}	55.5796	0.382156	newly	62.	42.69	{16., 80.}	49.7258	0.311005	Reff	1.84629	1.05918	{0.702474, 1.34008}	42.906	ar	889.	746.77	{474., 981.}	21.0562
11	R0	{1.99168, 8.13555}	{1.81459, 13.3577}	{{1.1315, 6.16453}, {2.65407, 18.9032}}	{26.0036, 74.1775}	inf	65.	87.6945	{6.80391, 208.373}	92.8078	0.454167	newly	78.	48.9	{5., 107.}	55.1026	0.48731	Reff	1.67899	1.25334	{0.814482, 1.91401}	32.5278	ar	772.	628.71	{237., 899.}	28.0661
12	R0	{1.79304, 9.18941}	{1.75857, 12.936}	{{1.07662, 6.62607}, {2.73737, 19.0443}}	{29.3975, 52.3856}	inf	28.	71.6597	{5.87089, 168.25}	193.5	0.493764	newly	33.	41.6	{4., 94.}	87.7576	0.428215	Reff	1.69442	1.35151	{0.894697, 2.09254}	29.2621	ar	725.	595.9	{172., 908.}	32.9297
13	R0	{1.84016, 18.3431}	{1.73752, 15.0353}	{{1.22382, 7.85712}, {2.39579, 20.}}	{22.5688, 21.7038}	inf	89.	91.1852	{32.0071, 159.015}	55.3512	0.258032	newly	37.	40.63	{16., 76.}	49.5405	0.203457	Reff	1.48685	0.975065	{0.617125, 1.35511}	36.8746	ar	762.	770.44	{534., 1016.}	19.9921
14	R0	{1.94221, 9.85971}	{1.77476, 13.1984}	{{1.11461, 6.78013}, {2.46533, 19.0269}}	{23.9498, 47.2941}	inf	111.	82.8701	{10.1501, 180.951}	58.2236	0.467283	newly	83.	41.89	{9., 85.}	53.3373	0.499721	Reff	1.44306	1.14203	{0.759278, 1.69433}	32.8973	ar	805.	624.85	{246., 889.}	27.1441
15	R0	{1.78578, 12.0541}	{1.69335, 13.7022}	{{1.23846, 7.34722}, {2.2419, 19.9193}}	{20.5243, 35.8619}	inf	101.	76.0513	{18.1138, 146.477}	46.8218	0.314191	newly	70.	35.65	{13., 59.}	52.4143	0.381128	Reff	1.39648	0.884161	{0.533468, 1.23197}	37.5696	ar	732.	758.41	{500., 978.}	21.2691
16	R0	{1.94463, 16.5603}	{1.73409, 12.9149}	{{1.11164, 6.0993}, {2.65407, 18.8228}}	{26.4851, 26.1199}	inf	94.	76.2558	{8.1986, 202.343}	67.162	0.508017	newly	42.	41.7	{5., 97.}	67.7143	0.407464	Reff	1.60626	1.25073	{0.814482, 1.72673}	28.6116	ar	757.	586.55	{202., 899.}	31.6499
17	R0	{2.08901, 16.4314}	{1.59947, 12.9291}	{{1.07874, 5.71232}, {2.21411, 19.0269}}	{27.4498, 28.7634}	inf	110.	62.8291	{2.41434, 147.459}	59.7035	0.58828	newly	50.	31.99	{3., 69.}	53.58	0.500125	Reff	1.69628	1.06456	{0.720352, 1.53048}	38.7814	ar	826.	555.11	{194., 827.}	34.161
18	R0	{2.76419, 18.56}	{1.7901, 13.0655}	{{1.07662, 6.0993}, {2.5458, 17.9172}}	{35.6491, 30.504}	inf	102.	82.2177	{7.99636, 195.555}	62.6517	0.496463	newly	42.	45.35	{4., 97.}	70.8333	0.424641	Reff	2.30257	1.25708	{0.807409, 1.74375}	45.9206	ar	920.	626.82	{214., 894.}	32.0283
19	R0	{2.3821, 17.1399}	{1.80892, 12.6911}	{{1.11164, 6.16453}, {2.64373, 18.2127}}	{28.9943, 29.1873}	inf	70.	75.999	{5.36887, 187.755}	78.191	0.483725	newly	39.	44.93	{3., 94.}	76.1795	0.463515	Reff	2.06528	1.36001	{0.894697, 2.0572}	36.0774	ar	883.	623.79	{172., 893.}	30.2661
20	R0	{2.78314, 19.6127}	{1.74766, 13.3882}	{{1.25007, 7.07053}, {2.37143, 19.3141}}	{37.6364, 32.0937}	inf	270.	87.7825	{25.702, 168.324}	67.8804	0.61188	newly	81.	42.22	{16., 82.}	51.6049	0.371305	Reff	1.35817	0.944595	{0.622222, 1.29769}	31.9894	ar	943.	783.23	{539., 999.}	19.9417
21	R0	{1.85124, 14.6794}	{1.65876, 13.3149}	{{1.11365, 6.16288}, {2.29738, 19.0355}}	{23.0598, 27.8774}	inf	54.	64.656	{4.38824, 147.459}	88.552	0.475723	newly	27.	32.47	{2., 67.}	79.8889	0.429591	Reff	1.61058	1.06417	{0.653976, 1.53048}	37.6074	ar	730.	578.13	{194., 855.}	29.3274
22	R0	{2.1028, 14.9852}	{1.78626, 13.1716}	{{1.11228, 6.62607}, {2.46533, 18.6082}}	{24.6982, 24.6366}	inf	146.	88.8864	{5.42087, 198.27}	56.1635	0.550474	newly	80.	43.97	{7., 86.}	50.9875	0.49887	Reff	1.46565	1.06077	{0.743452, 1.65342}	35.3407	ar	840.	621.34	{241., 889.}	28.7167
23	R0	{1.86601, 10.2157}	{1.77407, 13.12}	{{1.07662, 6.0993}, {2.65407, 17.9172}}	{24.9616, 44.2166}	inf	53.	79.8541	{7.99636, 195.555}	111.744	0.487037	newly	50.	42.86	{4., 96.}	62.48	0.45651	Reff	1.64022	1.26032	{0.813514, 1.74375}	29.4904	ar	747.	616.95	{214., 897.}	28.6814
24	R0	{2.34032, 10.0992}	{1.80679, 12.6776}	{{1.11228, 6.62607}, {2.64334, 17.9172}}	{28.101, 40.7121}	inf	45.	73.1984	{5.36887, 168.25}	114.057	0.448266	newly	41.	43.13	{4., 90.}	70.3659	0.437959	Reff	2.12969	1.37552	{0.852404, 2.0885}	36.443	ar	887.	634.51	{208., 899.}	29.3292
25	R0	{1.91548, 15.3401}	{1.70911, 13.458}	{{1.11228, 6.0993}, {2.47514, 18.9813}}	{24.4077, 25.7813}	inf	62.	75.2999	{2.48733, 170.225}	86.6833	0.519231	newly	31.	38.54	{1., 84.}	82.3871	0.478623	Reff	1.66455	1.14878	{0.768961, 1.59714}	35.4656	ar	765.	602.85	{244., 883.}	28.1346
26	R0	{2.75283, 10.6721}	{1.79681, 13.4913}	{{1.07195, 7.29751}, {2.59755, 17.5057}}	{35.5166, 38.871}	inf	42.	87.4616	{4.99197, 202.343}	156.172	0.515089	newly	48.	45.23	{3., 95.}	63.4792	0.451731	Reff	2.52985	1.24859	{0.814482, 1.72673}	51.0526	ar	926.	622.37	{188., 898.}	32.9104
27	R0	{2.08367, 19.7849}	{1.71949, 14.3982}	{{1.24903, 8.15092}, {2.34438, 20.}}	{24.3088, 27.5284}	inf	122.	81.7912	{24.3759, 154.567}	49.3167	0.32807	newly	56.	40.15	{15., 68.}	40.8036	0.256231	Reff	1.4669	1.07429	{0.753914, 1.40427}	30.0372	ar	833.	790.79	{577., 1008.}	16.6399
28	R0	{2.64334, 17.1765}	{1.78054, 13.0354}	{{1.06501, 6.8071}, {2.51417, 18.2464}}	{33.8255, 26.9708}	inf	150.	89.0716	{6.87127, 196.669}	57.9827	0.562726	newly	88.	44.78	{3., 92.}	53.9091	0.546754	Reff	1.90056	1.12995	{0.749697, 1.62684}	42.107	ar	934.	623.06	{216., 900.}	33.3148
29	R0	{2.87008, 13.3872}	{1.645, 14.338}	{{1.22609, 7.60971}, {2.16456, 20.}}	{42.7751, 29.3601}	inf	74.	75.5195	{25.0035, 149.574}	58.8445	0.271262	newly	61.	37.68	{14., 70.}	48.6885	0.332574	Reff	2.46827	1.13067	{0.740518, 1.44693}	54.1919	ar	941.	739.78	{489., 982.}	23.0861
30	R0	{2.06105, 19.6275}	{1.75046, 14.2555}	{{1.23695, 7.77108}, {2.39189, 20.}}	{22.315, 28.0492}	inf	138.	82.818	{15.2294, 180.136}	53.4976	0.423296	newly	44.	36.2	{10., 74.}	47.2727	0.288569	Reff	1.05732	0.819529	{0.476279, 1.14215}	28.283	ar	747.	794.31	{535., 1000.}	21.5301
31	R0	{2.30764, 13.435}	{1.84884, 12.9492}	{{1.11927, 6.31117}, {2.77502, 18.4249}}	{27.387, 27.0619}	inf	103.	87.85	{7.99636, 202.343}	66.8969	0.512065	newly	60.	47.91	{4., 102.}	60.0167	0.47152	Reff	1.88303	1.26516	{0.771241, 1.80429}	35.5897	ar	877.	648.48	{225., 917.}	27.7993
32	R0	{2.30565, 11.9237}	{1.81371, 12.7445}	{{1.11927, 6.0993}, {2.73737, 18.4249}}	{28.0166, 32.3357}	inf	62.	82.3467	{4.99197, 202.343}	96.9905	0.490482	newly	41.	44.9	{3., 97.}	73.7073	0.442253	Reff	1.97825	1.25197	{0.771241, 1.80429}	38.6568	ar	853.	636.35	{225., 899.}	28.0434
33	R0	{2.24031, 18.582}	{1.74786, 14.2389}	{{1.25248, 8.00125}, {2.40112, 19.8988}}	{27.8862, 25.5815}	inf	83.	93.5027	{26.72, 180.402}	62.4853	0.274794	newly	35.	45.3	{16., 78.}	62.0571	0.23014	Reff	1.90874	1.12526	{0.654026, 1.58668}	42.2517	ar	864.	780.6	{519., 1018.}	19.3681
34	R0	{2.33306, 15.9759}	{1.76896, 13.291}	{{1.08887, 6.77341}, {2.46533, 18.743}}	{27.977, 25.2043}	inf	138.	83.2573	{5.20499, 174.116}	55.8441	0.555721	newly	70.	41.82	{3., 91.}	51.1714	0.507379	Reff	1.80112	1.24045	{0.845712, 1.78306}	35.6189	ar	854.	608.94	{179., 898.}	30.6276
35	R0	{2.48357, 18.3573}	{1.75364, 12.9634}	{{1.11164, 6.0993}, {2.73737, 19.0443}}	{32.7348, 30.9169}	inf	114.	77.687	{8.1986, 202.343}	61.0661	0.527325	newly	58.	42.18	{5., 90.}	57.1724	0.446454	Reff	1.98686	1.25839	{0.843719, 1.7415}	38.5474	ar	835.	601.31	{203., 899.}	31.3066
36	R0	{2.59548, 19.9046}	{1.70529, 13.4813}	{{1.1298, 5.72868}, {2.24606, 19.0269}}	{35.8587, 32.2707}	inf	110.	77.3576	{2.41434, 167.585}	57.8025	0.539855	newly	53.	37.43	{3., 72.}	50.3208	0.466394	Reff	2.02967	1.05838	{0.654667, 1.53048}	48.8304	ar	863.	599.13	{201., 863.}	31.8806
37	R0	{2.29641, 16.9868}	{1.64099, 13.689}	{{1.20401, 7.0183}, {2.27664, 20.}}	{30.9599, 28.0108}	inf	149.	76.5639	{14.2649, 175.016}	58.5555	0.497272	newly	59.	34.02	{11., 67.}	49.322	0.380123	Reff	1.61208	0.859293	{0.551504, 1.11977}	46.6966	ar	877.	734.91	{513., 969.}	21.1505
38	R0	{2.65743, 15.4569}	{1.75248, 13.5501}	{{1.11461, 5.62019}, {2.43839, 19.1994}}	{35.049, 26.3439}	inf	119.	84.7198	{6.0459, 184.583}	56.5452	0.502083	newly	62.	40.74	{5., 83.}	49.9677	0.459784	Reff	1.96118	1.06676	{0.663115, 1.5528}	46.5455	ar	897.	615.52	{233., 885.}	31.835
39	R0	{2.67953, 14.5132}	{1.80293, 13.4882}	{{1.07195, 7.29751}, {2.60465, 17.5057}}	{34.107, 21.5672}	inf	36.	88.4472	{4.99197, 211.507}	188.856	0.532901	newly	28.	45.51	{3., 96.}	111.393	0.463021	Reff	2.47857	1.24181	{0.813756, 1.72673}	50.396	ar	894.	623.14	{188., 905.}	31.047
40	R0	{1.94594, 11.928}	{1.7349, 12.2447}	{{1.13176, 6.62607}, {2.46976, 17.2836}}	{23.8632, 28.0141}	inf	81.	86.4173	{8.54634, 190.36}	66.3027	0.405122	newly	45.	45.61	{8., 83.}	56.7778	0.350295	Reff	1.35827	0.992509	{0.641285, 1.3696}	31.0973	ar	796.	618.1	{245., 875.}	26.9673
41	R0	{1.66297, 10.0047}	{1.65172, 15.0508}	{{1.21307, 8.15824}, {2.22393, 20.}}	{20.1929, 58.36}	inf	49.	83.1411	{29.5877, 160.94}	91.2568	0.268755	newly	37.	37.71	{14., 67.}	44.0811	0.206155	Reff	1.32539	1.00295	{0.638738, 1.27818}	27.7153	ar	716.	750.12	{490., 998.}	21.9413
42	R0	{2.91568, 16.4453}	{1.82182, 12.7226}	{{1.10693, 6.18069}, {2.7811, 17.9391}}	{37.6525, 27.103}	inf	83.	79.906	{5.36887, 188.089}	71.3418	0.492053	newly	52.	45.95	{4., 98.}	60.8269	0.464325	Reff	2.50748	1.35406	{0.894697, 2.0885}	46.284	ar	919.	620.24	{204., 903.}	32.8009
43	R0	{2.55898, 15.8889}	{1.7614, 13.2879}	{{1.07469, 6.8071}, {2.51417, 17.6755}}	{32.7789, 22.9481}	inf	88.	79.8655	{4.99197, 191.076}	69.4709	0.498162	newly	64.	42.79	{3., 96.}	56.5469	0.498792	Reff	2.22119	1.26344	{0.814482, 1.83304}	43.6968	ar	904.	613.92	{213., 890.}	32.469
44	R0	{2.28649, 15.9017}	{1.73494, 13.1331}	{{1.12977, 6.0993}, {2.52651, 19.0269}}	{29.3433, 26.0883}	inf	132.	76.692	{5.40768, 175.555}	58.3465	0.576697	newly	68.	37.6	{7., 83.}	53.0588	0.522605	Reff	1.71487	1.14357	{0.743452, 1.67013}	37.8906	ar	880.	605.9	{231., 889.}	32.1841
45	R0	{2.65121, 18.6339}	{1.76898, 12.7282}	{{1.09005, 6.31117}, {2.73737, 17.9172}}	{35.1467, 32.2552}	inf	87.	78.8982	{5.36887, 187.755}	67.9181	0.483944	newly	50.	45.46	{3., 98.}	61.96	0.45582	Reff	2.3092	1.30747	{0.857811, 2.00473}	43.6261	ar	928.	606.4	{208., 910.}	34.7931
46	R0	{2.52291, 18.0251}	{1.76587, 13.6351}	{{1.14345, 6.0993}, {2.67299, 19.0531}}	{32.7523, 26.8993}	inf	129.	79.4762	{5.7877, 180.951}	59.6301	0.586993	newly	55.	39.68	{5., 91.}	55.0545	0.496093	Reff	1.99814	1.19602	{0.768961, 1.67839}	42.8773	ar	894.	612.88	{244., 904.}	32.1812
47	R0	{2.22618, 17.1225}	{1.78274, 13.1661}	{{1.12977, 6.55128}, {2.59602, 18.6082}}	{26.8528, 26.4247}	inf	75.	81.2457	{3.85453, 204.627}	77.2265	0.50382	newly	39.	41.14	{4., 96.}	66.3077	0.444117	Reff	1.91451	1.21223	{0.771954, 1.77856}	40.9061	ar	850.	609.56	{190., 902.}	30.88
48	R0	{2.04405, 16.073}	{1.815, 13.2545}	{{1.08887, 6.31117}, {2.53075, 18.9813}}	{25.1604, 26.1326}	inf	64.	89.2727	{6.60572, 204.627}	101.877	0.489129	newly	41.	43.89	{5., 96.}	69.5854	0.434706	Reff	1.69452	1.19674	{0.779198, 1.78306}	36.1848	ar	779.	627.93	{214., 907.}	28.7997
49	R0	{2.84396, 18.097}	{1.82057, 13.0191}	{{1.1298, 6.83219}, {2.51743, 18.4249}}	{36.1135, 29.5676}	inf	134.	74.5242	{4.16493, 200.111}	61.4232	0.608056	newly	69.	42.93	{4., 95.}	55.6087	0.548964	Reff	2.18132	1.36882	{0.833158, 2.02903}	38.0654	ar	952.	635.44	{234., 893.}	33.2521
50	R0	{1.85341, 18.0975}	{1.90311, 13.5338}	{{1.07662, 7.40677}, {2.79269, 18.7523}}	{25.0898, 26.8116}	inf	138.	105.058	{10.1286, 214.872}	52.9432	0.455277	newly	56.	51.31	{4., 98.}	53.0179	0.378603	Reff	1.27329	1.10595	{0.618628, 1.70448}	29.6091	ar	754.	679.71	{234., 910.}	23.6963
